# Contrast-enhanced spectral mammography: what is the 'added value' in a symptomatic setting? Initial findings from a UK centre

**DOI:** 10.1186/bcr3776

**Published:** 2015-11-05

**Authors:** Sarah Tennant, Eleanor Cornford, Jonathan James, Helen Burrell, Lisa Hamilton, Yan Chen

**Affiliations:** 1 Nottingham University Hospitals NHS Trust, Nottingham, UK; 2 Loughborough University, Leicester, UK

## Introduction

Contrast-enhanced spectral mammography (CESM) is a new technology. Dual energy acquisitions during one exposure yield two sets of images: a low energy (LE) set, equivalent to standard full field digital mammography (FFDM); and a recombined set displaying contrast uptake. In our symptomatic breast service, specific patients, including those with a P4/5 clinical abnormality are offered CESM instead of FFDM. Despite encouraging data from Europe and the USA, there are, until now, no UK data to support its use in this setting.

## Methods

Retrospective multi-reader review of 50 consecutive patients undergoing CESM. Anonymised LE images were reviewed and given a score for suspicion of malignancy. At least 3 weeks later, the entire examination (LE and recombined images) was reviewed. Pathology data were obtained for all cases. Differences in performance were assessed using receiver-operative characteristic (ROC) analysis. Sensitivity, specificity and lesion size (vs. MRI or histopathology) were analysed using a two-way independent *t *test.

## Results

Fifty females, mean age 49. Thirty-four (68 %) patients had biopsy-proven malignancy, 16 (32 %) were benign. CESM was more sensitive than LE alone (94 % vs. 86 %, *p* <0.025), more specific than LE alone (84 % vs. 63 %, *p* <0.025) and showed better size estimation (mean size difference 23 % vs. 31 %, *p* <0.025). ROC analysis showed CESM performance was better than LE alone (AUC 0.933 vs. 0.848, *p* <0.05).

## Conclusion

This preliminary study demonstrates the additional clinical utility of CESM in symptomatic patients. Its potential use in other clinical settings (e.g. screening of high-risk women) requires evaluation.

